# Method of Designing a Friction-Based Wedge Anchorage System for High-Strength CFRP Plates

**DOI:** 10.3390/ma14216443

**Published:** 2021-10-27

**Authors:** Wanxu Zhu, Wei Wei, Fengrong Liu, Rong Zeng

**Affiliations:** 1 College of Civil and Architecture Engineering, Guilin University of Technology, Guilin 541004, China; zhuwanxu@vip.163.com (W.Z.); vvkitty1997@163.com (W.W.); 2 Collaborative Innovation Center for Exploration of Hidden Nonferrous Metal Deposits and Development of New Materials in Guangxi, Guilin 541004, China; Fengrong.liu@glut.edu.cn; 3 Guangxi Engineering Research Center of Intelligent Structural Material, Guilin University of Technology, Guilin 541004, China; 4 Guangxi Key Laboratory of Geotechnical Mechanics and Engineering, Guilin University of Technology, Guilin 541004, China

**Keywords:** CFRP plate, friction-based anchor, anchorage mechanism, anti-slip, stress redistribution

## Abstract

The cables of high-strength carbon fiber reinforced polymer (CFRP) plates are starting to be applied to large spatial structures. However, their main anchorage systems rely on the adhesive force, which entails risks to their integrity resulting from aging of the binding agent. In this study, a friction-based wedge anchorage system was designed for CFRP plates. The working mechanism of the proposed anchorage system was explored both theoretically and experimentally. The anti-slip mechanism and condition of CFRP plates were formulated so that the equivalent frictional angle of the contact surface between a CFRP plate and wedges must not be smaller than the sum of the dip angle of the wedge external conical surface and the frictional angle between the wedges and barrel. An analysis of the stress distribution in the anchorage zone of the CFRP plate was conducted using the Tsai-Wu failure criterion, which concluded that the compressive stresses should be reduced on the section closer to the load-bearing end of the anchorage system. Furthermore, the anchorage efficiency coefficient was proposed, which depends on stress concentration coefficients, plate thickness, length of anchorage zone, dip angle of wedge external conical surface, and its frictional angle. Then, it was determined that the minimum length of an anchorage zone for the CFRP plates with various specifications should be at least 49 times larger than the CFRP thickness. A finite element analysis and static tensile tests on six specimens were carried out. The experimental results revealed that the anchorage efficiency coefficient of the optimized anchor reached 97.9%.

## 1. Introduction

Carbon Fiber Reinforcement Polymers (CFRPs) have excellent corrosion resistance, light weight, and high strength. They can also be conveniently used in construction [1,2]. CFRP cables have been successfully applied in long-span suspension and cable-stayed bridges [3,4], and have become the potential alternative to traditional steel cables [5,6]. Currently, their applications are being extended to large spatial structures [7]. Compared to the composite cables with a circular cross-section, cables with a square cross-section have a larger perimeter and thus a larger anchoring bonding area per unit length [8], which is beneficial for achieving effective anchoring.

While CFRP plates have the potential to be used in high-tension cables, their effective anchorage is a precondition for this type of application [9]. However, the transverse shear strength and compressive strength of CFRP are only about 1/10 of its longitudinal tensile strength [10], and, anchorage failure can easily be caused by even slight stress concentration inside the anchorage system because there is no plastic reserve [11,12]. Therefore, existing studies on anchorage systems for CFRP plates, conducted by both Chinese and foreign researchers, mostly intended to reduce the peak clamping stress and increase the anchoring force [13]. For instance, Wu et al. [14] studied concrete beams reinforced with plate with a press fit-bond anchor. Xian et al. [15] and Li et al. [16] developed a bonded plate anchor with wedge grooves, which demonstrated a superior performance. Zhu et al. [17] developed a wedge-type bonded anchor, whose efficiency coefficient exceeded 90%, and performed a systematic study of its application for beam reinforcement in practical engineering applications. However, the above anchorage methods still rely primarily on using binding agents or need an auxiliary binding agent for anchorage [18]. Thus, the durability of the binding agent has become a key factor influencing the anchorage performance [19]. By exploring the durability of CFRP anchor bolts, Zhu et al. [20] discovered that the bond-slip amount increased due to corrosion. From a fatigue test of a CFRP cable in water, Yang et al. [21] found that water accelerated the stress redistribution inside the anchor. The bonding medium experienced an aging failure caused by the environment and loading, thus the long-term reliability of the anchorage system was uncertain. In comparison to the anchorage methods that use binding agents, adopting mechanical anchorages, which utilize friction between the barrel and the CFRP plate, enables long-term efficient exploitation of the high-strength characteristic of CFRP plates [22]. Ghafoori et al. [23,24] developed a plate press fit-type anchor that did not require any binding agent for strengthening of steel girders. However, their anchorage system was complex and cumbersome in field applications. Ibaraki Univ. [25,26] proposed an anchorage method that combines flat plate anchorage and U type CFRP sheets, and concrete reinforcement tests were conducted, but the tension magnitude of CFRP plate is still restricted at a low level. Ye et al. [27,28] and Mohee et al. [29,30] placed a tapered wedge inside a plate press fit-type anchor and achieved favorable anchorage performance without using any binding agent to clamp the CFRP plates. However, this anchorage system was not convenient for engineering applications due to its large size. Moreover, the internal stress redistribution mechanisms in the anchor have not been systematically explored in the existing studies, and there is still lack of a theoretical foundation for anchor optimization.

In order to facilitate wider application of CFRP plates in civil engineering, a friction-based wedge anchor for CFRP plates is proposed in this study and its design method is systematically developed. The proposed anchorage structure uses mechanical friction and biting force, which eliminates the problem of aging-related binder failure of traditional anchorages. Furthermore, the proposed anchorage structure is small, safe, and convenient in construction, which will aid its practical applications. The optimal design of the anchorage is studied considering slip resistance, stress redistribution, and factors influencing the anchorage efficiency to determine the key dimensions of anchorage device. Finite element simulations and static tension tests were also carried out. In the latter, a 50 × 3 mm CFRP plate anchorage was tested and validated, and the next step will be to conduct tests on other commonly used CFRP plate anchorages.

## 2. Theoretical Analysis

**Figure 1:**
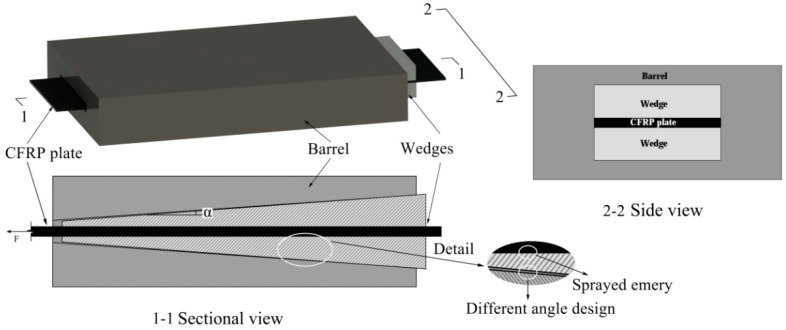
Schematic diagram of the friction-based wedge anchorage system.

**Figure 2:**
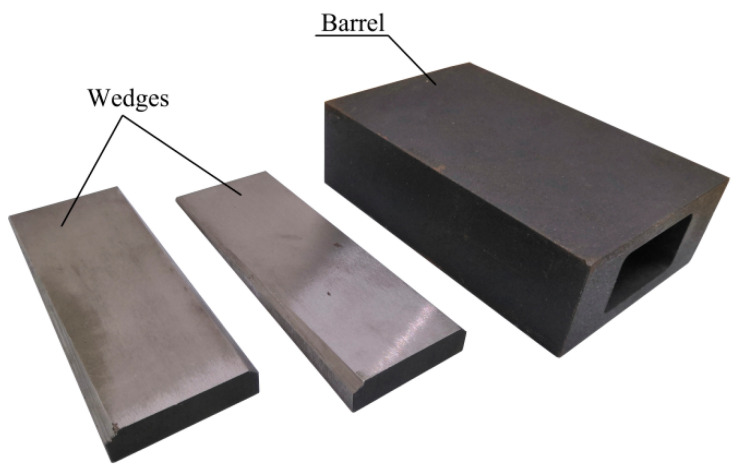
Parts of the friction-based wedge anchor.

A diagram of the proposed anchorage system is shown in Figure 1 and its constituent parts are shown in Figure 2. The friction-based wedge anchor for CFRP plates comprises two parts: the barrel and the wedges. When the CFRP plate is subjected to a longitudinal tensile force, the wedges are pulled into the barrel by the frictional force between them and the CFRP plate, and the anchor generates a force that clamps the CFRP plate tightly. 

The failure of a wedge-type anchor for CFRP plates can generally be of three types [31]: slip failure, pinch-off failure, and CFRP plate tensile failure. The wedge-type anchor can experience slip failure or CFRP plate pinch-off failure due to excessive relative displacement or compressive stress in the CFRP plates in the anchorage zone. On the other hand, the CFRP plates are susceptible to tensile failure when both longitudinal displacement and compressive stress are uniformly distributed within a certain zone, providing an ideal anchoring effect. Hence, in friction-based wedge anchorage design the emphasis should be on analyzing the influences of design parameters on the relative displacement and compressive stress in CFRP plates in the anchorage zone, so that the CFRP plates can be clamped tightly without excessive damage.

### 2.1. Anti-Slip Mechanism

The friction-based wedge anchor had two key contact interfaces. The contact interface between the barrel and the wedge is referred to as Interface 1, while that between the CFRP plate and wedge as Interface 2. The wedges are required to self-lock after the CFRP plate is tensioned so that retraction does not occur when the tensile force in the CFRP plate decreases, thus the following should be satisfied:

(1) α<β

where *α* and *β* are the taper and frictional angle of wedge external conical surface, respectively.

**Figure 3:**
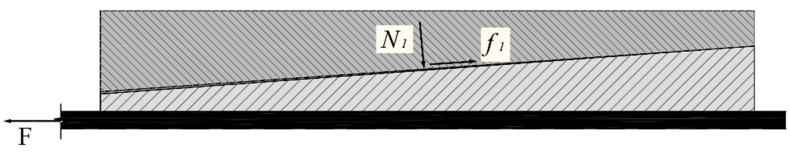
Force diagram of Interface 1.

After wedge self-locking, the anchorage system can be treated in the analysis as a plane-symmetric model (Figure 3). The equilibrium of forces requires the following to hold:

(2) $$F=N_{1}\sin\alpha +f_{1}\cos\alpha$$

where *F* is the tensile force in CFRP plate, and *f*_1_ and *N*_1_ are the frictional and normal force acting on Interface 1, respectively. It can be demonstrated that:

(3) $$F=N_{1}\tan\left(\alpha +\beta \right)$$

**Figure 4:**
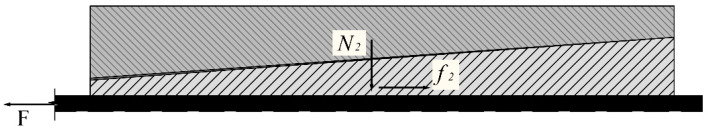
Force diagram of Interface 2.

To ensure that the CFRP plate does not slip along its contact interfaces with the wedges, the frictional resistance generated by the wedges and the CFRP plate should be greater than the tensioning force in the CFRP plate. A free-body diagram for the CFRP plate can be analyzed considering only the frictional resistance between the CFRP plate and the wedges, leading to the following condition (Figure 4):

(4) $$F\leq \mu N_{2}$$

where *μ* is the frictional coefficient of Interface 2, and *f*_2_ and *N*_2_ are the frictional and normal forces acting on Interface 2, respectively.

Combining Equations (3) and (4), the following relationship can be obtained:

(5) μ≥tan(α+β)

If μ=tanθ, where *θ* is the equivalent frictional angle of the contact interface between CFRP plate and wedges, then:

(6) θ≥α+β

### 2.2. Stress Redistribution Mechanism

The tensile and compressive stress distributions inside the anchor in safe state were obtained according to the relevant strength theories, and the stress redistribution mechanism was further elucidated to guide the design of the anchorage system. The strength theories commonly adopted for composite materials include maximum stress theory, maximum strain theory, Tsai-Hill theory, and Tsai-Wu theory [32,33]. The Tsai-Wu strength theory [34], which considers the difference in tensile and compressive strengths, can better reflect the failure of carbon fiber materials. It is assumed that the failure surface can be expressed as follows:

(7) $$F_{i}\sigma_{i}+F_{ij}\sigma_{i}\sigma_{j}=1,\;\;\;\;\;\;\;i,j=1,2\ldots ,\;6$$

When the CFRP plate is in a linear elastic state, the left-hand side of the above equation must not exceed 1. In a planar stress state, the expression for orthotropic material can be transformed into the following form:

(8) $$F_{11}\sigma_{1}^{2}+F_{22}\sigma_{2}^{2}+F_{66}\sigma_{6}^{2}+F_{1}\sigma_{1}+F_{2}\sigma_{2}+F_{6}\sigma_{6}+2F_{12}\sigma_{1}\sigma_{2}+2F_{16}\sigma_{1}\sigma_{6}+2F_{26}\sigma_{2}\sigma_{6}\leq 1$$

where *σ*_1_, *σ*_2_, and *σ*_6_ are the longitudinal, transverse, and shear stress, respectively. Coefficients *F_i_* and *F_ij_*, which are related to the strength of CFRP plate material, can be obtained from experimental data according to the following formulas:

(9) $$\{\begin{array}{c} F_{1}=\frac{1}{X_{t}}-\frac{1}{X_{c}},\;F_{11}=\frac{1}{X_{t}X_{c}}\\ F_{2}=\frac{1}{Y_{t}}-\frac{1}{Y_{c}},\;F_{22}=\frac{1}{Y_{t}Y_{c}},\;F_{12}=\frac{1}{2\sigma_{m}^{2}}\left[1-\left(\frac{1}{X_{t}}-\frac{1}{X_{c}}+\frac{1}{Y_{t}}-\frac{1}{Y_{c}}\right)\sigma_{m}-\left(\frac{1}{X_{t}X_{c}}+\frac{1}{Y_{t}Y_{c}}\right)\sigma_{m}^{2}\right]\\ F_{16}=F_{16}=F_{6}=0,\;\;\;F_{66}=\frac{1}{S^{2}} \end{array}$$

where *X_t_* and *X_c_* are the longitudinal tensile and compressive strength, respectively, *Y_t_* and *Y_c_* are the transverse tensile and comprehensive strength, respectively, and *S* is the shear strength. The high-strength CFRP plates investigated in this study were formed through the pultrusion process, and the carbon fiber yarns were parallel and uniformly distributed in the longitudinal direction. Therefore, *X_t_* was determined based on the strength of carbon fiber yarns, and it was taken as *X_t_* = 2400 MPa according to the yarns strength grade adopted in engineering applications. The compressive and tensile strengths of CFRP plate in other directions were obtained from the corresponding values for the isotropic epoxy resin matrix. It was experimentally determined that *X_c_* = *Y_t_* = *Y_c_* = 120 MPa [35], and then *F_2_* = 0. Strength *X_c_* of the CFRP plate was much smaller than *X_t_*, thus the limiting value of two-way tensile failure test mainly depended on the ultimate compressive strength; thus, $\sigma \;\approx \;-X_{c}$. Shear stress *σ*_6_ = 0, because the longitudinal and transverse directions of CFRP plate were essentially identical with the directions of the principal stresses in the anchor. Using *σ*_6_ = 0, the following simplified formulas were obtained:

(10) $$F_{1}\sigma_{1}+F_{11}\sigma_{1}{}^{2}+F_{22}\sigma_{2}{}^{2}+2F_{12}\sigma_{1}\sigma_{2}\leq 1$$

(11) $$\left(\frac{1}{X_{t}}-\frac{1}{X_{c}}\right)\sigma_{1}+\frac{1}{X_{t}X_{c}}\sigma_{1}{}^{2}+\frac{1}{Y_{t}Y_{c}}\sigma_{2}{}^{2}+\frac{\sigma_{1}\sigma_{2}}{X_{c}{}^{2}}\left[1-\left(\frac{1}{X_{t}}-\frac{1}{X_{c}}+\frac{1}{Y_{t}}-\frac{1}{Y_{c}}\right)X_{c}-\left(\frac{1}{X_{t}X_{c}}+\frac{1}{Y_{t}Y_{c}}\right)X_{c}{}^{2}\right]\leq 1$$

It was assumed that $\xi \;=\;X_{t}/X_{c}$ , and the clamping damage coefficient was defined as $\eta \;=\;\sigma_{2}/X_{c}$ and substituted to obtain the following:

(12) $$\frac{1}{\xi X_{c}{}^{2}}\left[\sigma_{1}{}^{2}+(1-\xi +\eta \xi )X_{c}\sigma_{1}+(\xi \eta^{2}-\xi )X_{c}{}^{2}\right]\leq 0$$

(13) $$\sigma_{1}\leq X_{c}\left(\sqrt{\frac{{\left(1-\xi -\eta \xi \right)}^{2}}{4}+\xi \left(1-\eta^{2}\right)}-\frac{1-\xi -\eta \xi }{2}\right)$$

(14) $$\sigma_{1}\leq 0.05X_{t}\left(\sqrt{\frac{{\left(19+20\eta \right)}^{2}}{4}+20\left(1-\eta^{2}\right)}+\frac{19+20\eta }{2}\right),\;\eta \in \left[-1,0\right],\;\sigma_{1}\in \left[0,2400\right]$$

The expressions were simplified using MATLAB, and then the relationship between the tensile stress and compressive stress of the CFRP plate in the anchorage zone in safe state was obtained as follows:

(15) $$\sigma_{1}-19.06\sigma_{2}\leq X_{t}$$

**Figure 5:**
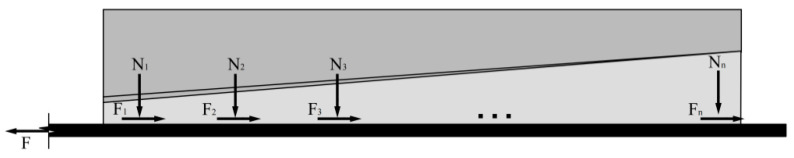
Internal force diagram of anchor.

Starting from the load-bearing end, the anchor was divided into *n* segments along its length. Each section of the CFRP plate was subjected to a vertical normal force, *N_i_*, and longitudinal frictional resistance, *F_i_*, as shown in Figure 5.

Given that any nonuniform contact between the wedges and the CFRP plate will lead to a concentration of longitudinal tensile stress and vertical compressive stress, the following notation is adopted:

(16) $$\sigma_{1}=k_{i1}{\bar{\sigma }}_{1i},\;\sigma_{2}=k_{i2}{\bar{\sigma }}_{2i},\;i=1,2\ldots ,n$$

where ${\bar{\sigma }}_{1i}$ and ${\bar{\sigma }}_{2i}$ are the average tensile and compressive stresses in segment *i* of CFRP plate, respectively, and *k_i1_* and *k_i2_* are the tensile and compressive stress concentration coefficients of segment *i* of CFRP plate, respectively.

According to Equation (15), the relationship between tensile and compressive stress of each segment in safe state should satisfy the following:

(17) $$k_{i1}{\bar{\sigma }}_{1i}-19.06k_{i2}{\bar{\sigma }}_{2i}\leq X_{t}$$

Due to the frictional forces between the wedge and the CFRP plate, the longitudinal stress in the CFRP plate will be gradually reduced moving away from the load-bearing end. The average longitudinal tensile stress in segment *i* of the CFRP plate can be expressed as follows:

(18) $${\bar{\sigma }}_{1i}=\frac{F}{S_{i}}-\frac{F_{1}}{S_{i}}-\frac{F_{2}}{S_{i}}-\cdots -\frac{F_{i-1}}{S_{i}}=\frac{F-\sum_{j=1}^{i-1}F_{j}}{S_{i}}$$

where *S* is the cross-sections area of segment *i* (*i* =1,2…,*n*) of the CFRP plate.

The stress concentration coefficients, *k*_1_ and *k*_2_, of all sections are assumed to be equal, and then:

(19) $$k_{1}{\bar{\sigma }}_{1i}-19.06k_{2}{\bar{\sigma }}_{2i}\leq X_{t}$$

Under the ideal state, the stress concentration coefficients remain the same in each segment thus for any section, the following holds true:

(20) $$k_{1}{\bar{\sigma }}_{1i}-19.06k_{2}{\bar{\sigma }}_{2i}=k_{1}{\bar{\sigma }}_{1j}-19.06k_{2}{\bar{\sigma }}_{2j},\;i,j=1,2\ldots ,n$$

The ultimate stress in the stress concentration zone on each section is required to be equal, and it was substituted into Equation (20) to obtain the following:

(21) $$k_{1}\frac{F-\sum_{m=1}^{i-1}F_{m}}{S}-19.06k_{2}\frac{N_{i}}{S_{h}}=k_{1}\frac{F-\sum_{m=1}^{j-1}F_{m}}{S}-19.06k_{2}\frac{N_{j}}{S_{h}}$$

where *S* is the sectional area of the CFRP plate (*S = bd*, in which *b* is the width and *d* stands for the thickness of the CFRP plate), and *S_h_* is the contact area between the CFRP plate segment and the wedges (*S_h_ = 2bL/n*, in which *L* is the wedge length). The formulas for the areas were substituted into the above equation to obtain:

(22) $$k_{1}\frac{F-\sum_{m=1}^{i-1}F_{m}}{bd}-19.06k_{2}\frac{nN_{i}}{2bL}=k_{1}\frac{F-\sum_{m=1}^{j-1}F_{m}}{bd}-19.06k_{2}\frac{nN_{j}}{2bL}$$

(23) $$k_{1}\left(F-\sum_{m=1}^{i-1}F_{m}\right)-9.53k_{2}\frac{ndN_{i}}{L}=k_{1}\left(F-\sum_{m=1}^{j-1}F_{m}\right)-9.53k_{2}\frac{ndN_{j}}{L}$$

To simplify the formula, it was assumed that $k_{2}^{\prime }$ = 9.53*k*_2_ · *n* · *d*/*L*, and then:

(24) $$k_{1}\left(F-\sum_{m=1}^{i-1}F_{m}\right)-k_{2}^{\prime }N_{i}=k_{1}\left(F-\sum_{m=1}^{j-1}F_{m}\right)-k_{2}^{\prime }N_{j}$$

As segments closer to the load-bearing end have greater values of expression $F-\sum_{m=1}^{j-1}F_{m}$, the following can be concluded:

(25) $$N_{n}>N_{n-1}>\ldots >N_{1}$$

It can thus be concluded that the clamping stress of sections closer to the load-bearing end should be smaller, and a suitable compressive stress adjustment mechanism should make it possible for the wedges to gradually press the CFRP plate tightly from the large end to the small end.

### 2.3. Factors Influencing Anchorage Efficiency Coefficient

From Equations (19) and (24), the following can be deduced:

(26) $$\sum_{i=1}^{n}k_{1}{\bar{\sigma }}_{1i}S_{i}-19.06\sum_{i=1}^{n}k_{2}{\bar{\sigma }}_{2i}S_{i}\leq n\cdot X_{t}\cdot S\;k_{1}\sum_{i=1}^{n}\left(F-\sum_{j=1}^{i-1}F_{j}\right)+k_{2}^{\prime }\sum_{i=1}^{n}N_{i}\leq nF_{b}\;k_{1}\sum_{i=1}^{n}F-k_{1}\sum_{i=1}^{n}\sum_{j=1}^{i-1}F_{j}+k_{2}^{\prime }\sum_{i=1}^{n}N_{i}\leq nF_{b}\;k_{1}\left(nF-\sum_{i=1}^{n}\sum_{j=1}^{i-1}F_{j}\right)+k_{2}^{\prime }\sum_{i=1}^{n}N_{i}\leq nF_{b}$$

where *F_b_* is the standard ultimate tensile force of the CFRP plate.

The frictional resistance, *F_i_*, of each segment was assumed to be proportional to the clamping force, *N_i_*, namely:

(27) $$F_{i}=kN_{i}a$$

where *k* is the proportionality coefficient, *k =* 2*tan(α*+*β)*. Then:

(28) $$\sum_{i=1}^{n}F_{i}=k\sum_{i=1}^{n}N_{i}$$

As $F=\sum_{i=1}^{n}F_{i}$, the following can be obtained:

(29) $$F=k\sum_{i=1}^{n}N_{i}$$

Hence:

(30) $$\begin{array}{ll} \sum_{i=1}^{n}\sum_{j=1}^{i-1}F_{j} & =\left(n-1\right)F_{1}+\left(n-2\right)F_{2}+\cdot \cdot \cdot 2F_{n-2}+F_{n-1}\\ & =\left(\frac{n-1}{2}\right)F_{1}+\left(\frac{n-2}{2}F_{2}+\frac{1}{2}F_{1}\right)+\left(\frac{n-3}{2}F_{3}+\frac{1}{2}F_{2}+\frac{1}{2}F_{1}\right)+\cdot \cdot \cdot +\sum_{i=1}^{n}\frac{1}{2}F_{i}\\ & =k\left[\left(\frac{n-1}{2}N_{1}\right)+\left(\frac{n-2}{2}N_{2}+\frac{1}{2}N_{1}\right)+\left(\frac{n-3}{2}N_{3}+\frac{1}{2}N_{2}+\frac{1}{2}N_{1}\right)+\cdot \cdot \cdot +\sum_{i=1}^{n}\frac{1}{2}N_{i}\right] \end{array}\;\sum_{i=1}^{n}\sum_{j=1}^{i-1}F_{j}\leq \frac{n-1}{2}k\sum_{i=1}^{n}N_{i}=\frac{n-1}{2}F$$

By substituting Equation (30) into Equation (26), the following is obtained:

(31) $$k_{1}F\left(n-\frac{n-1}{2}\right)+k_{2}^{\prime }\frac{F}{k}\leq nF_{b}$$

(32) $$\frac{F}{F_{b}}\leq \frac{n}{k_{1}\frac{n+1}{2}+\frac{k_{2}^{\prime }}{k}}$$

The relationship $k_{2}^{\prime }=9.53k_{2}\cdot n\cdot d/L$ was substituted into the above equation to obtain the following:

(33) $$\frac{F}{F_{b}}\leq \frac{n}{k_{1}\frac{n+1}{2}+9.53\frac{k_{2}dn}{Lk}}$$

If *n* is a large number, then

(34) $$\frac{F}{F_{b}}\leq \frac{2}{k_{1}+19.06\frac{k_{2}d}{Lk}}$$

When reaching the ultimate breaking force, *F_pk_*, of the anchorage system, *F* should still satisfy Equation (34).

The following relationships can be obtained using the anchorage efficiency coefficient, $\eta_{A}=F_{pk}/F_{b}$:

(35) $$\eta_{A}\leq \frac{2}{k_{1}+19.06\frac{k_{2}d}{Lk}}$$

(36) $$\eta_{A}\leq \frac{2}{k_{1}+9.53\frac{k_{2}d}{L\tan(\alpha +\beta )}}$$

The anchorage efficiency coefficient, *m_A_*, was defined as follows:

(37) $$m_{A}=\frac{2}{k_{1}+9.53\frac{k_{2}d}{L\tan(\alpha +\beta )}}$$

The following conclusions can be obtained from Equations (36) and (37):

(1) The anchorage efficiency index, *m_A_*, depends on the stress concentration coefficients (*k*_1_ and *k*_2_), plate thickness (*d*), length of anchorage zone (*L*), taper of wedge external conical surface (*α*), and its frictional angle (*β*). Larger values of *m_A_* correspond to better anchorage system performance.

(2) Smaller values of stress concentration coefficients *k*_1_ and *k*_2_ correspond to higher values of the anchorage system efficiency coefficient. In order to ensure high anchorage performance, the geometric parameters of the anchorage system should be selected such that *m_A_* exceeds 1.0 and *k*_1_ is not greater than 2.

(3) The greater the sum of the taper of wedge external conical surface (*α*) and frictional angle of wedge external surface (*β*), the higher the anchorage efficiency coefficient, but this is restricted by equation (6), namely, protecting the CFRP plate from slipping.

(4) The greater the thickness of CFRP plate (*d*), the longer the anchorage zone (*L*) required, and the two are in direct proportion to each other. Moreover, their ratio affects the anchorage system performance, and smaller ratios produce higher anchorage efficiency coefficients.

## 3. Determination of Parameters

The key geometric parameters of the anchorage system can be determined according to Equations (1), (6), (25) and (36) as follows:

(1) Determination of *α* and *β*

In general, the values of *α* and *β* of anchorage systems with different specifications in the same anchorage system are essentially identical. Using the frictional coefficient of contact interface under high stress determined by Huang [35], the frictional coefficient between the wedges and the barrel (steel block-steel plate) was determined to be about 0.24. Thus the frictional angle of the interface was *β =* 14°, which decreased to 7° after a lubricating agent was applied. To pull smoothly the wedge into the barrel, *α* should not be too large, and it was taken as 4°, which met the requirement of Equation (1).

(2) Determine the parameters of the contact interface between CFRP plate and wedges

In order to reduce the stress concentration coefficients (*k*_1_ and *k*_2_) as much as possible, emery was sprayed evenly on the clamping surfaces of the wedges instead of suing indentations or tooth, thus the damage caused to the CFRP plate was very small. Following Meng [36], in which the interfacial frictional coefficient of composite coating made of 0.5 mm emery was 0.72, the equivalent frictional angle of Interface 2 was taken as 35°, and the maximum sum of *α* and *β* was 18°. The non-slip requirements for the CFRP plate were already satisfied by Equation (6).

(3) Determination of *L*

The length of the anchorage zone should be as short as possible in order to reduce the size and weight of the anchor. The anchorage systems in the same system should be designed according to the same anchorage efficiency coefficient and stress concentration coefficient. As stipulated in the standard [37], the anchorage efficiency coefficient should be greater than or equal to 0.9. The stress distribution in the CFRP plate was assumed to be uniform in the anchorage zone. The minimum values of *k*_1_ and *k*_2_ were both taken as 1, and that of *β* as 7°, all of which were substituted into Equation (36) to obtain the minimum anchorage length of CFRP plate as follows:

(38) L≥49d

Furthermore, the anchorage lengths of the CFRP plate commonly used in engineering practice were determined as shown in Table 1 [38].

(4) Selection of barrel and wedge materials

The 40*Cr* steel has good toughness and high strength and was selected as the barrel material, because it will have to bear safely a large circumferential tensile stress. Its elongation ratio is greater or equal to 9%, the yield strength and ultimate tensile strength are 785 MPa and 980 MPa, respectively. The wedges should be made of soft steel in an effort to mitigate the stress concentration in the CFRP plate. To reduce cost, the wedges can be made of A3 steel or 45 steel without heat treatment. 

(5) Determination of contact parameters between barrel and wedges

The dip angle of wedge external conical surface was set to be slightly larger than that of the barrel, and the big ends of wedges were the first ones to contact the barrel so that the compressive stress distribution could meet the requirements of Equation (25). In general, the optimal performance was reached when the difference between the two angles was 10 min, which was adjusted according to the static loading test results.

## 4. Finite element analysis

The anchorage of a 50 × 3 mm CFRP plate was taken as an example for finite element analysis to obtain the stress distribution in the optimized anchor. Using symmetry, a 1/4-model of the anchorage device was formulated.

The material of the barrel was 40Cr steel, with elastic modulus of 210 GPa and Poisson’s ratio of 0.3. The material of the wedges was 45 steel, with an elastic modulus of 206 GPa and Poisson’s ratio of 0.3. The elastic modulus of the CFRP plate was 160 GPa, Poisson’s ratio 0.25, and standard strength 2,400 MPa. The friction coefficient between the barrel and wedges was assumed as 0.24 [35] and that between the wedges and CFRP plate as 0.72 [36].

**Figure 6:**
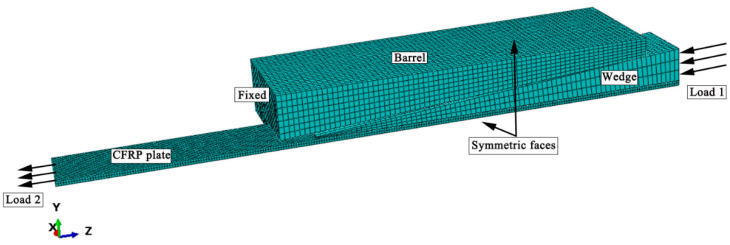
Finite element mesh.

The finite element mesh is shown in Figure 6. Since the purpose of the finite element analysis was to determine the stress concentration on the surface of the CFRP plate, the meshing of CFRP plate was finer than in the other components. The loading was divided into two steps. First, a preload stress of 100 MPa was applied to the wedge, and then it was unloaded. Next, a tensile load of 1,440 MPa (0.6Xt) was applied to the CFRP plate, while the displacement of the barrel in the longitudinal direction was restrained.

**Figure 7:**
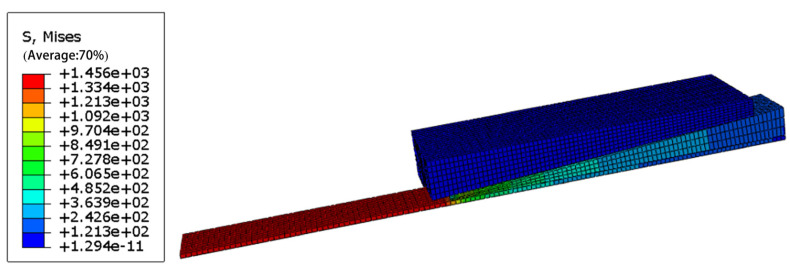
Von Mises stress distribution.

**Figure 8:**
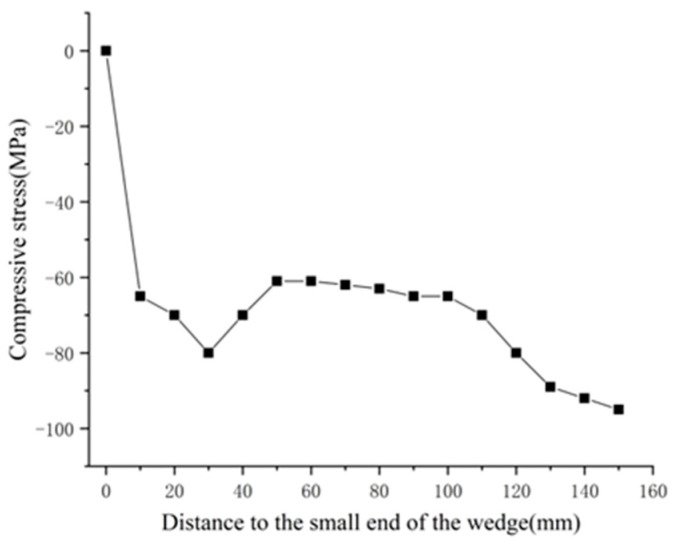
Compressive stress in CFRP plate vs. distance from small end of wedge.

Figure 7 and Table 2 show the simulation results, while the changes in the compressive stress in the CFRP plate with the distance from the small end of the wedge are shown in Figure 8.

The stress in the barrel was mainly concentrated at the end of the large hole where it contacts the wedge. The maximum stress in the wedge occurred also in the contact area with the barrel. The maximum von Mises stresses in both components were smaller than their yield strengths. Thus, the finite element analysis verified the correctness of material selection for the barrel and wedges to ensure appropriate safety margins, as discussed in Point (4) in Section 3.

As seen in Figure 8, except for a stress concentration in the contact area at the large end of the wedge, the remainder of the compressive stresses was in the range of 60~80 MPa. The compressive stress near the free end of the CFRP plate was greater than that at the loaded end, which indicates that the wedge with the dip angle assumed in Point (5) in Section 3 gradually compressed the CFRP plate from the large end to the small end, such that the clamping stresses in the sections closer to the load-bearing end were smaller, and the compressive stress distribution met the requirements stated in Equation (25).

## 5. Static Tensile Test

### 5.1. Experimental program

**Figure 9:**
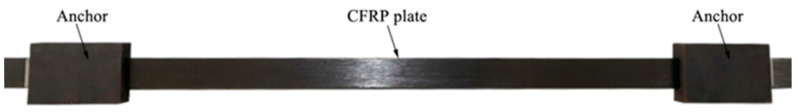
Assembled specimen.

The anchor was optimized for the CFRP plate with a strength of 2400 MPa and sectional area of 50 mm × 3 mm. The length of barrel was taken as 150 mm and the blanking length of CFRP plate as 1500 mm. The length of free section of the specimen was ensured to be greater than 1000 mm. Six CFRP plate anchors were assembled to perform a static tensile test. A specimen with the assembled anchor is shown in Figure 9.

**Figure 10:**
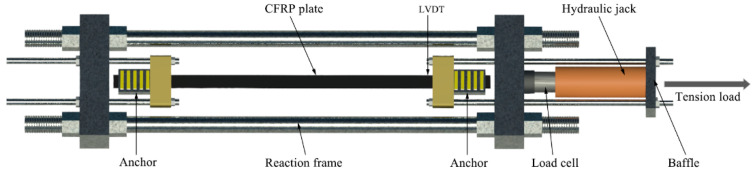
Schematic diagram of testing apparatus.

A schematic diagram of the testing apparatus is shown in Figure 10. The test was conducted in a laboratory horizontal reaction frame. One end of the specimen was fixed while the other end was pulled by a hydraulic jack. A load cell was used to measure the tensioning force. A displacement sensor (LVDT) was placed on the inner side of the tensioned end.

First, specimens were preloaded to 5% of *F_b_*. After straightening of the CFRP plate and confirming the apparatus was working correctly, the specimens were unloaded and the initial readings were recorded. The main loading test followed the phased multi-stage loading protocol: The multi-stage loading was conducted using 5% of *F_b_* in the initial phase. After the load reached 60% of *F_b_*, the multi-stage loading was implemented according to 2% of *F_b_*. When the load reached 70% of *F_b_*, it was held for 5 min, and then increased continuously until the specimen failed. The loading rate was controlled within 0.05–0.07 mm/s.

### 5.2. Results and Discussion

**Figure 11:**
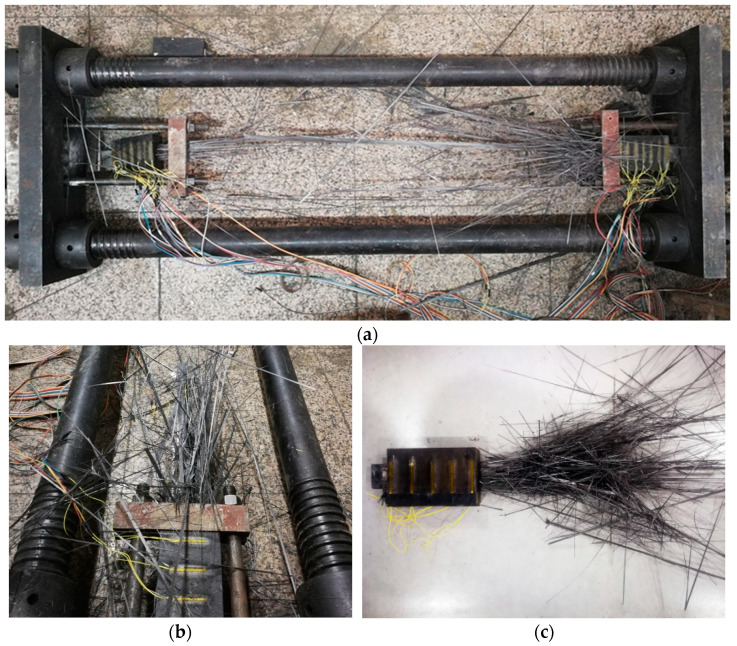
Typical fracture forms of CFRP plates. (**a**) testing apparatus, (**b**) detail view of one end of the testing apparatus, (**c**) anchorage.

Each specimen suffered tensile fracture. As the load increased, the CFRP plates started cracking from the middle towards both sides, then damage gradually extended to the anchors, and plates disintegrated into individual carbon fiber yarns in a sudden explosion. Each failed specimen presented the characteristic burst shape. The tensile failure of specimens was always due to breaking of carbon fiber yarns, as shown in Figure 11.

**Figure 12:**
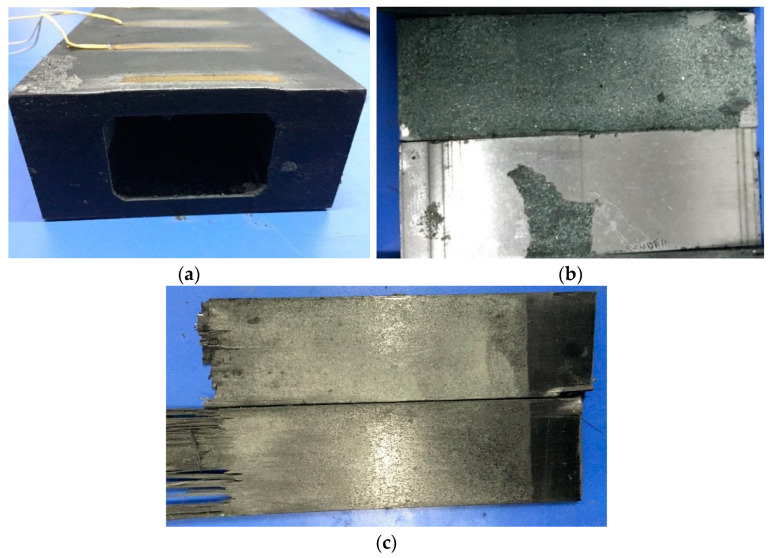
Anchorage after testing. (**a**) Barrel after testing, (**b**) Wedge after testing, (**c**) CFRP plate after testing.

After the test, the wedges were withdrawn, and the barrel, wedges, and CFRP plate were inspected. The barrel and wedges were intact, as shown in Figure 12. Thus, it was confirmed that the 40Cr steel had sufficient toughness to be used for the barrels as no expansion or cracking damage occurred to the barrels. The barrel material met the strength requirement with a wide safety margin, which confirmed the correctness of decisions made in Point (5) in Section 3 and the results of the finite element analysis.

The emery sprayed on the wedges was cleaned, which revealed the bite marks left on the surface by squeezed emery, as shown in Figure 12b.

There were obvious signs of extension and emery biting into the surface of the anchorage area of the CFRP plates, as shown in Figure 12c. These were mainly distributed over the area where the large end of the wedge contacted the CFRP plate. There were, however, no obvious signs of uneven contact. This demonstrates that the adopted anchorage design distributed the pressure evenly over the CFRP plate. During tensioning, some of the emery between the contact surface of the wedges and the CFRP plate gradually bit into the surfaces of both, but the degree of mechanical damage to the CFRP plate was small.

The results of the static tensile test are summarized in Table 3. The test results showed that irrespective of whether lubricating was applied to the external conical surfaces of the wedges or not, the anchorage system always met its performance requirements, with the anchorage efficiency coefficient reaching as high as 97.9%. However, the anchorage efficiency coefficient of specimens whose Interface 2 was coated with lubricant was improved, indicating that the wedges were pulled more easily into the barrel when adding lubricant, as proposed in Point (1) in Section 3. The stress concentration in the anchorage zone was small thanks to the uniform deformation state.

**Figure 13:**
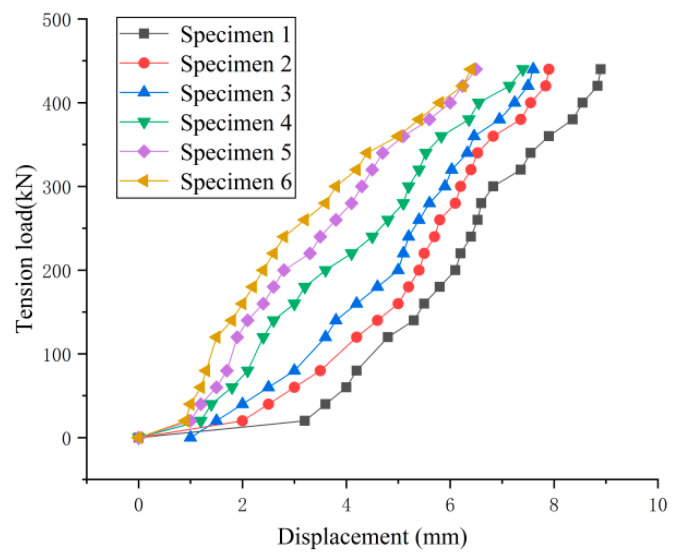
Force-displacement curves.

To investigate the effect of the wedge on the CFRP plate during tensioning, the force- displacement curves were plotted for the six experimental specimens in Figure 13. (For safety, the LVDT was removed after the load reached a certain value, thus full force-displacement curves are not available).

The force-displacement curves of the six specimens were consistent with one another. During the loading process, the displacements of the CFRP plates increased linearly and rapidly with the load without sudden changes, which indicated that the stiffness of the CFRP plates did not change significantly during the tensioning process, and no slip occurred. This shows that by the suitable selection of parameters *α*, *β* and *L* in Points (1) and (3) in Section 3 it is possible to achieve no-slip requirements for the CFRP plates, utilize fully their strength, and ensure the anchoring efficiency.

**Figure 14:**
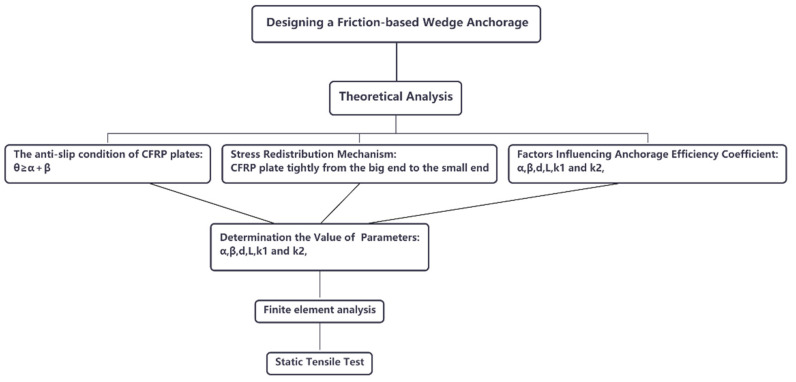
The flow chart of the analysis process.

Except for Specimen 1, in the remaining specimens adding lubricant reduced the friction coefficient between the barrel and the wedge, and the displacement of Specimen 1 was larger than the rest of the specimens. This further verified the assumption made in Point (1) in Section 3 that reducing the friction coefficient between the barrel and the wedge would facilitate pulling the wedges into the barrel, thus reducing the displacement of the CFRP plate and improving the anchorage performance. In addition, a flow chart of the analysis process is presented in Figure 14.

## 6. Conclusions

In this study, a design method for a friction-based wedge anchorage system for high-strength CFRP plates was systematically developed and investigated. An anti-slip condition for the anchor assembly of CFRP plates was derived. Then, the factors influencing anchorage performance and their effect were analyzed using the Tsai-Wu failure criterion. Mathematical expressions for the relationships between the anchorage efficiency coefficient and the influencing factors were derived, and the key geometric parameters of the anchorage system were determined. A finite element analysis and a static tensile test were performed to better prove the proposed method soundness. The following conclusions were drawn:

(1) The equivalent frictional angle of the contact interface between the CFRP plate and wedges should not be smaller than the sum of the dip angle of the wedge external conical surface and the frictional angle between wedges and barrel, to prevent the CFRP plate from slipping out of the anchorage system.

(2) The compressive stress of the section closer to the load-bearing end of the anchorage system should be reduced. The dip angle of wedge external conical surface should be slightly larger than that of the barrel, with the angle difference of 10 min. In this way, the wedges can clamp the CFRP plate tightly from the large end to the small end and improve the anchorage performance.

(3) The anchorage efficiency coefficient, *m_A_*, is determined by the stress concentration coefficients (*k_1_* and *k_2_*), plate thickness (*d*), length of anchorage zone (*L*), taper of wedge external conical surface (*α*), and its frictional angle (*β*). The optimal design of an anchor for CFRP plates can be achieved using the design expressions. The minimum anchorage length of CFRP plates with various specifications was determined as *L* ≥ 49*d*.

(4) The results of the static tensile test of anchor assemblies for CFRP plates demonstrated that all optimally designed specimens satisfied the anchorage requirements. The wedges can be easily pulled into the barrel under the low-stress state after a lubricating agent is applied to their interfaces, which can further increase the anchorage efficiency coefficient (up to as high as 97.9%). It was verified through the tests that the optimally designed anchor provides reliable and efficient anchorage.

## Figures and Tables

**Table 1:** Anchorage lengths of CFRP plate.

Width of CFRP Plate (mm)	Thickness of CFRP Plate (mm)	Minimum Anchorage Length (mm)
50	1.2	67
	2	98
	3	147
	4	196

**Table 2:** Finite element analysis results.

Component	Maximum von Mises stress (MPa)
Barrel	694.12
Wedge	573.20
CFRP plate	1456.33

**Table 3:** Static loading test results.

Specimen No.	Lubricant on Interface 2	Failure Mode	Failure Stress of Carbon Plate(MPa)	Anchorage Efficiency Coefficient
1	No	Breaking of fiber yarns	2256.0	0.940
2	Yes	Breaking of fiber yarns	2281.0	0.950
3	Yes	Breaking of fiber yarns	2300.5	0.958
4	Yes	Breaking of fiber yarns	2326.5	0.969
5	Yes	Breaking of fiber yarns	2313.0	0.964
6	Yes	Breaking of fiber yarns	2350.5	0.979

## Data Availability

All data, models, and code generated or used during the study appear in the submitted article.
